# The efficacy of intravaginal electrical stimulation (IVES) in treating female with urinary incontinence symptom from meta-analysis of nine randomized controlled trials

**DOI:** 10.3389/fneur.2022.933679

**Published:** 2022-09-13

**Authors:** Huibao Yao, Xiaofei Zhang, Fengze Sun, Gonglin Tang, Jitao Wu, Zhongbao Zhou

**Affiliations:** ^1^Department of Urology, The Affiliated Yantai Yuhuangding Hospital of Qingdao University, Yantai, China; ^2^Department of Neurology, The Affiliated Yantai Yuhuangding Hospital of Qingdao University, Yantai, China; ^3^Department of Urology, Beijing TianTan Hospital, Capital Medical University, Beijing, China

**Keywords:** intravaginal electrical stimulation, women, urinary incontinence, randomized controlled trials, meta-analysis

## Abstract

**Background:**

Urinary incontinence (UI) is a common disease in the middle-aged and elderly women, and physical therapy has gradually become the mainstream treatment of UI. We conducted a meta-analysis to evaluate the efficacy of intravaginal electrical stimulation (IVES) in the treatment of UI.

**Methods:**

From January 2006 to December 2021, we finally selected nine randomized controlled trials (RCTs) including 657 participants from PubMed, EMBASE, and Cochrane databases to evaluate the efficacy of IVES in the treatment of female UI. Continuous data were represented by mean difference and 95% CI, while dichotomous data were represented by odds ratio and 95% CI. All the data were analyzed by the Review Manager Version 5.4.

**Results:**

Compared with the control group, there were significant improvements in urine pad test (*P* = 0.01), urinary incontinence frequency (*P* = 0.04), some indicators in the incontinence quality of life questionnaire and King's health questionnaires, and subjective feeling of cure (*P* = 0.009) in the IVES group. However, in other indicators reflecting UI, there was no significant difference between the IVES group and the control group. In addition, subgroup analysis showed that IVES and IVES combined with training could significantly reduce the weight of the urine pad, which reflected the improvement of urine leakage.

**Conclusion:**

This meta-analysis proved that IVES can partially improve the symptoms of female patients with UI compared with the control group. However, it still needs to be further evaluated through more high-quality research in the future.

## Introduction

Urinary incontinence (UI) is a common disease, which is defined as the phenomenon of transurethral urine leakage that is not controlled by will by the International Conference Society (ICS) (1). Women are more likely to suffer from UI than men due to age growth, improper postpartum care, congenital urogenital abnormalities, and previous gynecological surgery (2–5). Although UI will not endanger the life of patients, long-term suffering from UI will seriously affect the physical and mental health, and the quality of life of patients. Patients will deliberately avoid going out, reduce social activities, and gradually become disconnected from society. Therefore, urinary incontinence is also known as “social cancer” (6–8).

Physical therapy is usually used as the first-line therapy of UI treatment, including pelvic floor muscle training, bladder training, intravaginal electrical stimulation (IVES), and other methods (9, 10). Intravaginal electrical stimulation (IVES) can inhibit the activity of the reflex parasympathetic nerve and reduce the involuntary contraction of the bladder detrusor (11, 12). Other studies have also shown that IVES stimulate pelvic floor muscle contraction and relaxation directly through the sensors in the vagina, increasing pelvic floor muscle strength, and effectively improving UI symptoms (13). However, due to embarrassment, discomfort, and other shortcomings, its clinical use rate is not high now (14).

We conducted a meta-analysis of the retrieved randomized controlled trials (RCTs) to evaluate the efficacy of IVES in the treatment of female urinary incontinence.

## Materials and methods

### Search strategy

We searched related literature from PubMed, Embase, and Cochrane databases according to the guidelines of Preferred Reporting Items for Systematic Reviews and Meta-Analysis (PRISMA) (15), and the retrieval time was limited from January 2006 to December 2021. We formulated the retrieval strategy according to the PICOS (populations, interventions, comparators, outcomes, and study designs) principle. The keywords searched are as follows: electrical stimulation, intravaginal electrical stimulation, female, women, incontinence, urinary incontinence, RCT, and randomized controlled trials. There were no restrictions on the language of the article when searching the literature. The two authors searched according to the search strategy, then compared the search results, respectively. All the articles retrieved were read independently by two researchers. In case of dispute, a third researcher would be invited to read and provide suggestions to reach an agreement. In addition, we would also download and read some references to related articles if necessary.

### Inclusion and exclusion criteria

All the articles included are supposed to meet the criteria as follows: (1) the articles describing IVES in treating women's urinary incontinence; (2) every article's content should be obtained and all the data are true and effective; (3) the experimental method is a randomized controlled trial. If the same research is published repeatedly in different journals or at different times, we would select the latest research literature for this meta-analysis. In addition, if an article type is a case report, review article, meeting report, or abstract, this article would be excluded. The relevant details of inclusion criteria and exclusion criteria are shown in Table 1.

**Table 1:** Search strategy according to populations, interventions, comparators, outcomes, and study designs (PICOS).

	**Population**	**Intervention**	**Comparator**	**Outcomes**	**Study design**
Inclusion criteria	Women with urinary incontinence	Intravaginal electrical stimulation	Placebo Training Other electrical stimulation	Pad test Bladder diary King's health questionnaire I-QOL questionnaire PFM strength Subjective feeling	Randomized Controlled Trials
Exclusion Criteria	Not performed	Not performed	Not performed	Not performed	Letters, comments, reviews, meeting reports

### Quality assessment

We evaluated all included randomized controlled trials studies according to the guideline of the *Cochrane Handbook for Systematic Reviews of Interventions v5.10*. At the same time, each article was classified according to the following three quality evaluation criteria: (+) low risk of bias, (?) moderate risk of bias or insufficient evidence to judge the degree of bias, and (–) high risk of bias. All the authors participated independently in the risk assessment of each RCT. After the evaluation was completed, everyone summarized and discussed until all the evaluation results were consistent.

### Data extraction

Two authors independently collected data from the included articles and tabulated them. The extracted data include (a) first author's name, (b) published year, (c) country, (d) sample size of experimental group and control group, (e) setup of experimental group and control group, (f) methodology, and (g) outcome indicators. This study did not need ethical approval since it was a retrospective analysis of published research.

### Statistical analyses

The data in this study were analyzed by Review Manager Version 5.4.0 (Cochrane Collaboration, Oxford, UK). We used fixed or random effect models to evaluate the indicators. The continuous data type was expressed by mean difference (MD) and 95% confidence interval (CI), while the dichotomous data type was expressed by odds ratio (OR) and 95% CI. In addition, we tested the heterogeneity by *Q*-value test or *I*^2^ test. If the *P* < 0.05 in the *Q*-value statistic test or *I*^2^ was greater than 50% in the *I*^2^ test, the study was considered to be heterogeneous and would be analyzed by the random effect model. Otherwise, the study would be analyzed by a fixed effect model because of homogeneity. In the results section, the data with *P*-value < 0.05 were considered statistically significant.

## Results

### Study selection, search results, and characteristics of the trials

**Figure 1:**
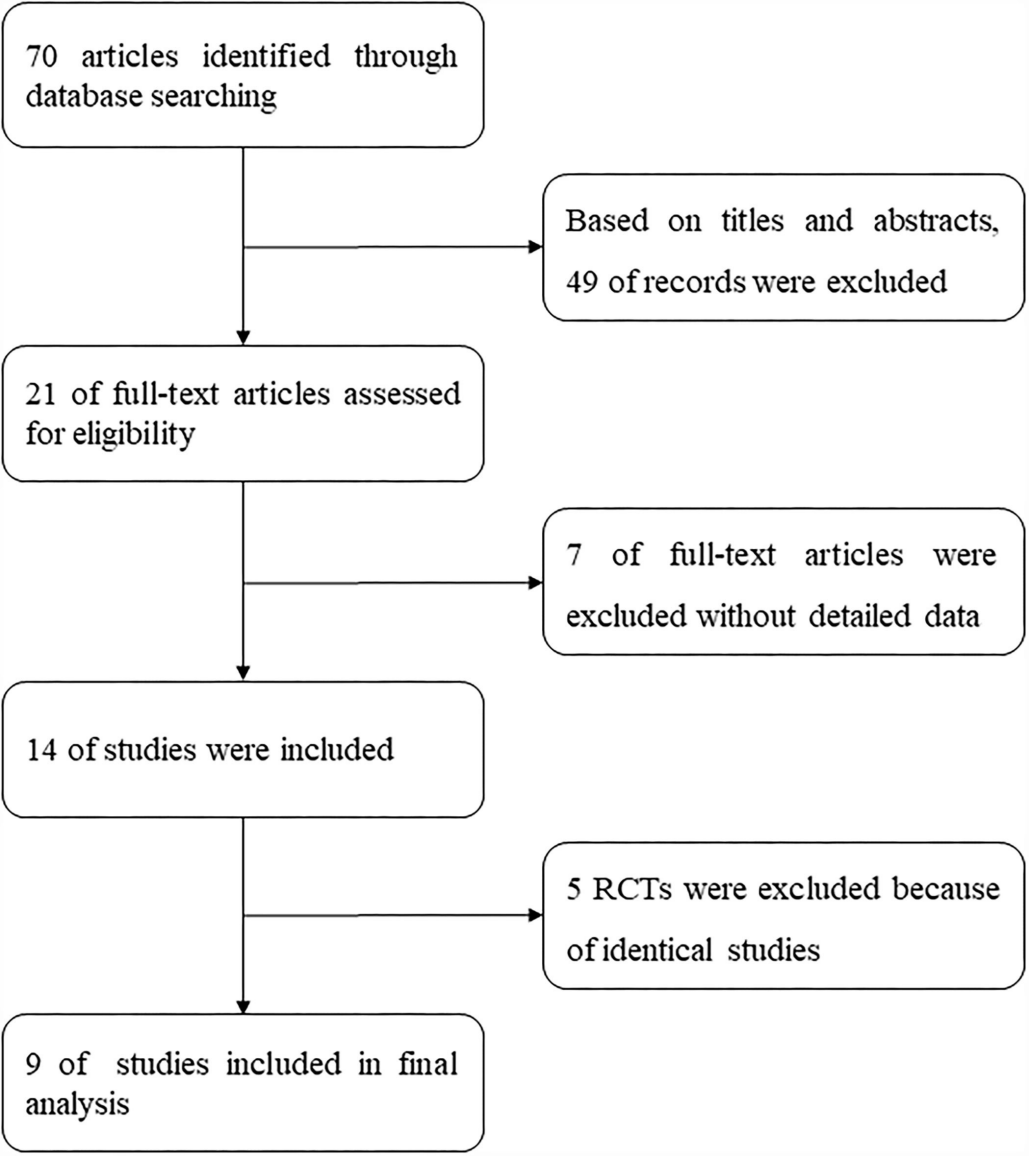
Flowchart of selection PRISMA. RCTs, randomized controlled trials.

We searched and finally found 70 articles according to the aforementioned search strategy and another 49 articles were deleted when we first screened the titles and abstracts. An additional seven articles, of the remaining 21 articles were excluded because they lacked relevant data. Five duplicate articles were excluded from the remaining 14 articles, and at last, nine randomized controlled trials were included in our study to evaluate the efficacy of IVES in improving UI symptoms (16–24). The process of study selection is presented in Figure 1, and the characteristics of these studies are shown in Table 2.

**Table 2:** Characteristics of all the studies.

**Author's name**	**Year**	**Country**	**Sample size**		**Setup**		**Methodology**	**Outcome**
			**Experimental group**	**Control group**	**Experimental group**	**Control group**		
Amaro et al. (18)	2006	Brazil	20	20	• electrical stimulator: Dualplex Uro 996 • frequency: 4 Hz • pulse width: 700 μs • site of action: vagina	• electrical stimulator: Dualplex Uro 996 • same type of vaginal probe without electrical energy • site of action: vagina	• 20 mins/time • 3 times/week • 7 weeks	• pad test • urodynamic study
Correia et al. (16)^a^	2013	Brazil	15	15	• electrical stimulator: Dualpex 961 (Quark Medical Products) • frequency: 50 Hz • pulse width: 700 μs • site of action: vagina	• electrical stimulator: Dualpex 961 (Quark Medical Products) • frequency: 50 Hz • pulse width: 700 μs • site of action: suprapubic region and ischial tuberosity	• 20 mins/time • 2 times/week • 6 weeks	• pad test • pelvic floor muscle strength • King's Health Questionnaire
Correia et al. (16)^b^	2013	Brazil	15	15		• No treatment		
Dmochowski et al. (21)	2019	America	91	89	• electrical stimulator: iTouch sure (TensCare Ltd, Epsom, Surrey, UK) • frequency: 50 Hz • pulse width: 300 μs • site of action: vagina	• electrical stimulator: INNOVO® (Atlantic Therapeutics, Galway, Ireland) • frequency: 50 Hz • pulse width: 620 μs • site of action: around the pelvic area	• 30 mins/time • 5 times/week • 12 weeks	• pad test • Incontinence Quality of Life • bladder diary
Elmelund et al. (24)	2018	Denmark	14	13	• pelvic floor muscle training • electrical stimulator: Cefar Peristim Pro® (NMKimport, Værløse, DK) • frequency: 40 Hz • pulse width: 250 μs • site of action: vagina	• pelvic floor muscle training	• 7 time/weeks • 12 weeks	• ICIQ-UI-SF • urethral pressure reflectometry • bladder diary • pad test
Franco et al. (17)	2011	Brazil	20	22	• electrical stimulator: Dualpex 961 (Quark Medical Products) • frequency: 10 Hz • pulse width: 700 μs • site of action: vagina	• electrical stimulator: Dualpex 961 (Quark Medical Products) • frequency: 20 Hz • pulse width: 200 μs • site of action: Plantar arch of left foot	• 30 mins/time • 1 time/week • 12 weeks	• Incontinence Quality of Life • SF-36 quality of life scale
Terlikowski et al. (22)	2013	Poland	64	29	• electrical stimulator: VeriProbe (Verity Medical) • frequency: 10–40 Hz • pulse width: 200–250 μs • 20 mins/time • site of action: vagina	• electrical stimulator: VeriProbe (Verity Medical) • frequency: 2 Hz • pulse width: 50 μs • site of action: vagina	• Experimental • 20 mins/time • 2 times/week • 8 weeks • Control • 2 seconds/time • 2 times/week • 8 weeks	• Incontinence Quality of Life • bladder diary • pad test
Ugurlucan et al. (19)	2013	Turkey	35	17	• electrical stimulator: Endomed-M 433 (Delf Instruments Physical Medicine B.V.) • frequency: 10–50 Hz • pulse width: 300 μs or 1 ms • maximal output current: 24–60 mA • site of action: vagina	• electrical stimulator: Urgent PC Neuromodulation System • frequency: 20 Hz • pulse width: 200 μs • site of action: 3–4 cm cephalad to the medial malleolus	• 20 mins/time • 3 times/week • 6–8 weeks	• bladder diary • pad test • pelvic floor muscle strength • King's Health Questionnaire • subjective evaluation
Wang et al. (20)	2017	China	40	80	• electrical stimulator: PHENIX USB 4 neuromuscular stimulation therapy system (Electronic Concept Lignon Innovation, Montpellier, France) • frequency: 12.5–30 Hz • site of action: vagina	• electrical stimulator: G6805-2 Multi-Purpose Health Device (Shanghai Medical Instruments High-Techno, Shanghai, China) frequency: 2 Hz • pulse width: 2 ms • site of action: sacrococcygeal joint and tip of the coccyx	• Experimental • 45 mins/time • 3 times/week • 4 weeks • Control • 60 mins/time • 3 times/week • 3 weeks	• pad number • subjective evaluation
Yildiz et al. (23)	2021	Turkey	29	29	• bladder training and • electrical stimulator: Enraf Nonius Myomed 632 • frequency: 10 Hz • pulse width: 100 ms • site of action: vagina	• bladder training	• 20 mins/time • 3 times/week • 8 weeks	• bladder diary • pad test • pelvic floor muscle strength • IIQ7 • OAB-V8 • subjective evaluation

a, IVES group vs. SESG group.

b, IVES group vs. sham group.

### Risk of bias

**Figure 2:**
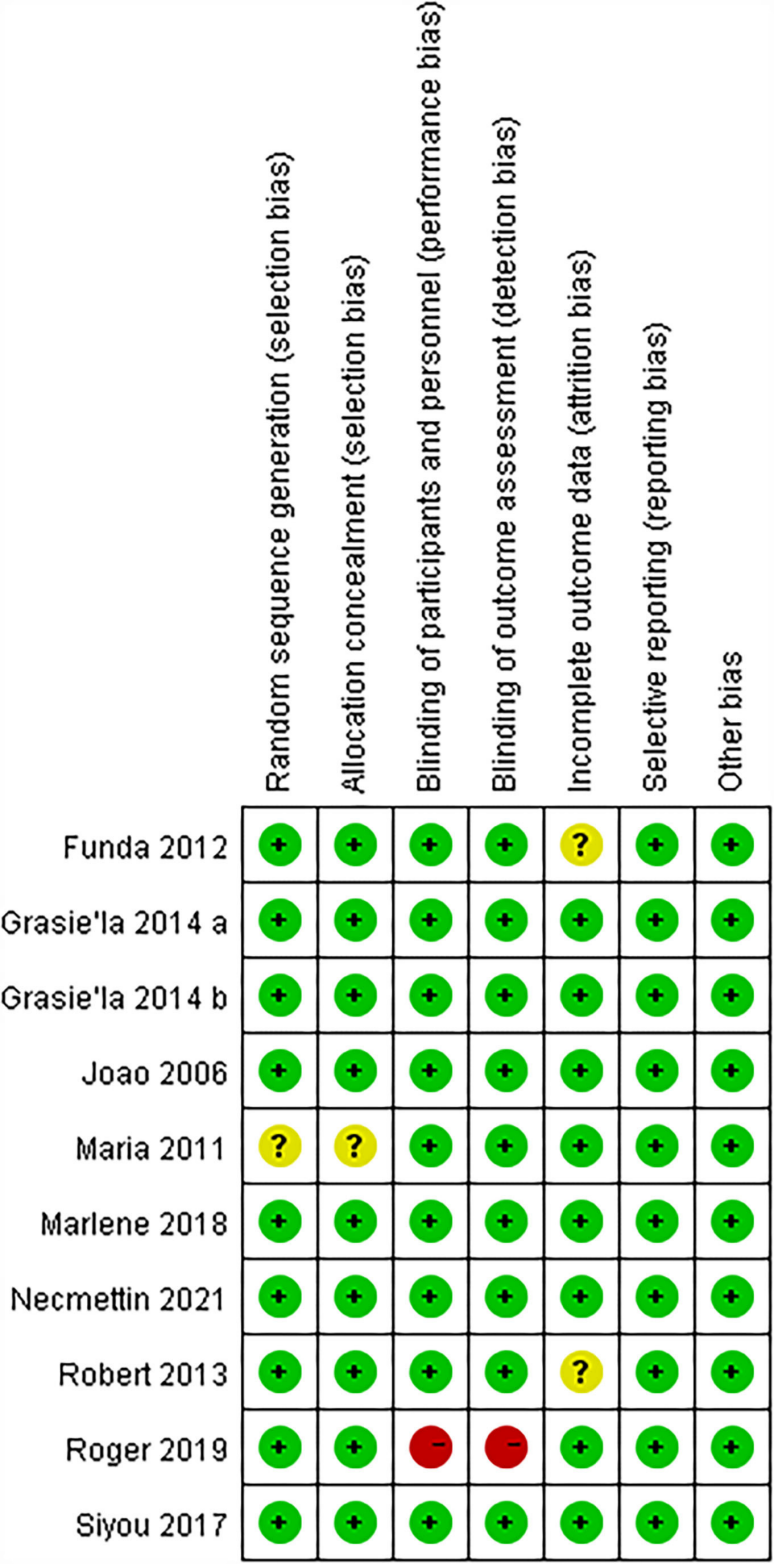
The risk of bias summary.

**Figure 3:**
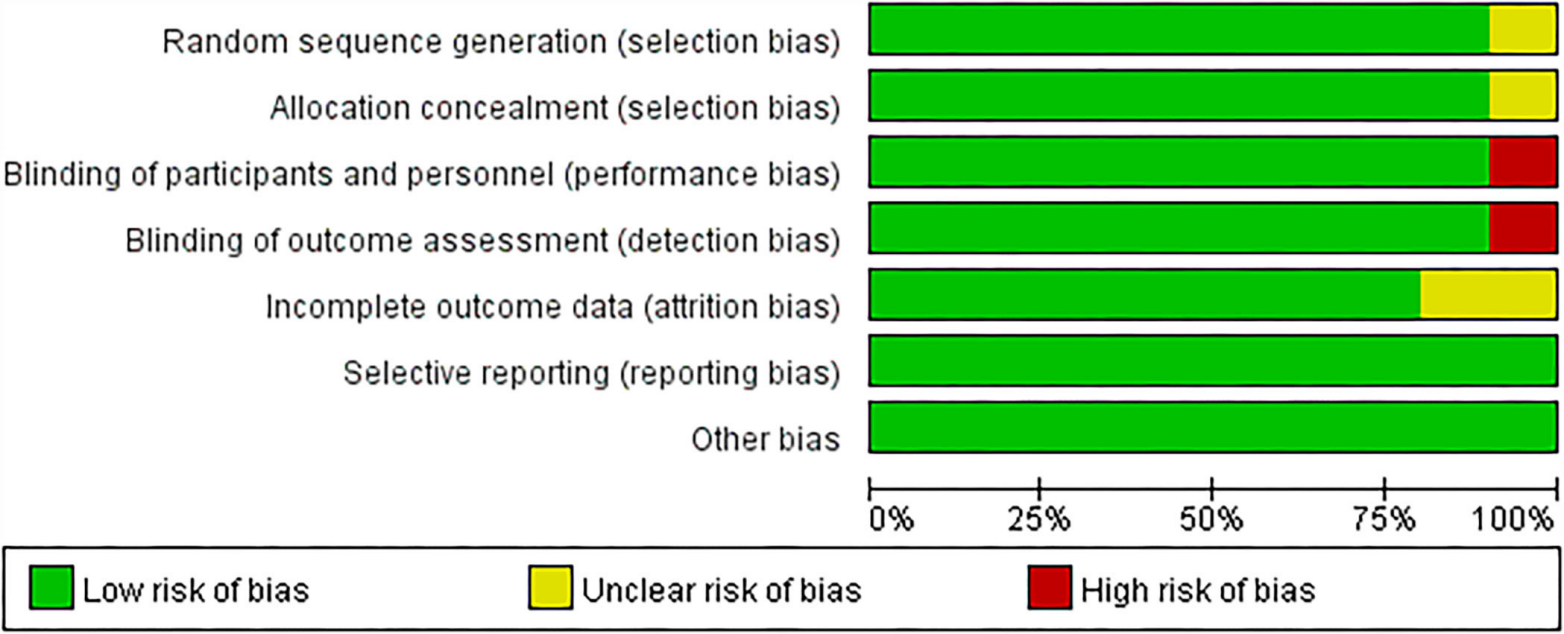
The risk of bias graph.

All included studies in this meta-analysis were randomized controlled trials. The summary and graph of bias risk are shown in Figures 2, 3.

### Pad test

**Figure 4:**
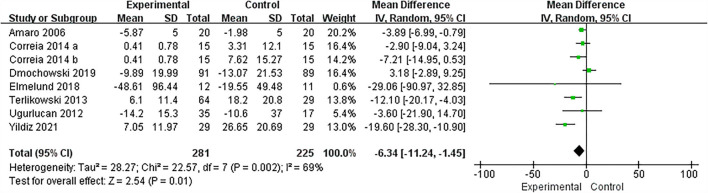
Forest plots showing the improvement of pad test by IVES treatment. SD, standard deviation; IV, inverse variance; CI, confidence interval; df, degrees of freedom.

The urine pad test of seven randomized controlled trials involving 491 patients (266 in the vaginal electrical stimulation group and 225 in the control group) showed that the weight (unit: g) of the urine pad in the vaginal electrical stimulation group decreased significantly (MD = −6.34; 95% CI = [−11.24, −1.45]; *P* = 0.01) compared with the control group and the difference was statistically significant (Figure 4). The data involved in the analysis adopts the change value or final value before and after processing.

### Bladder diary

#### Incontinence episodes

**Figure 5:**
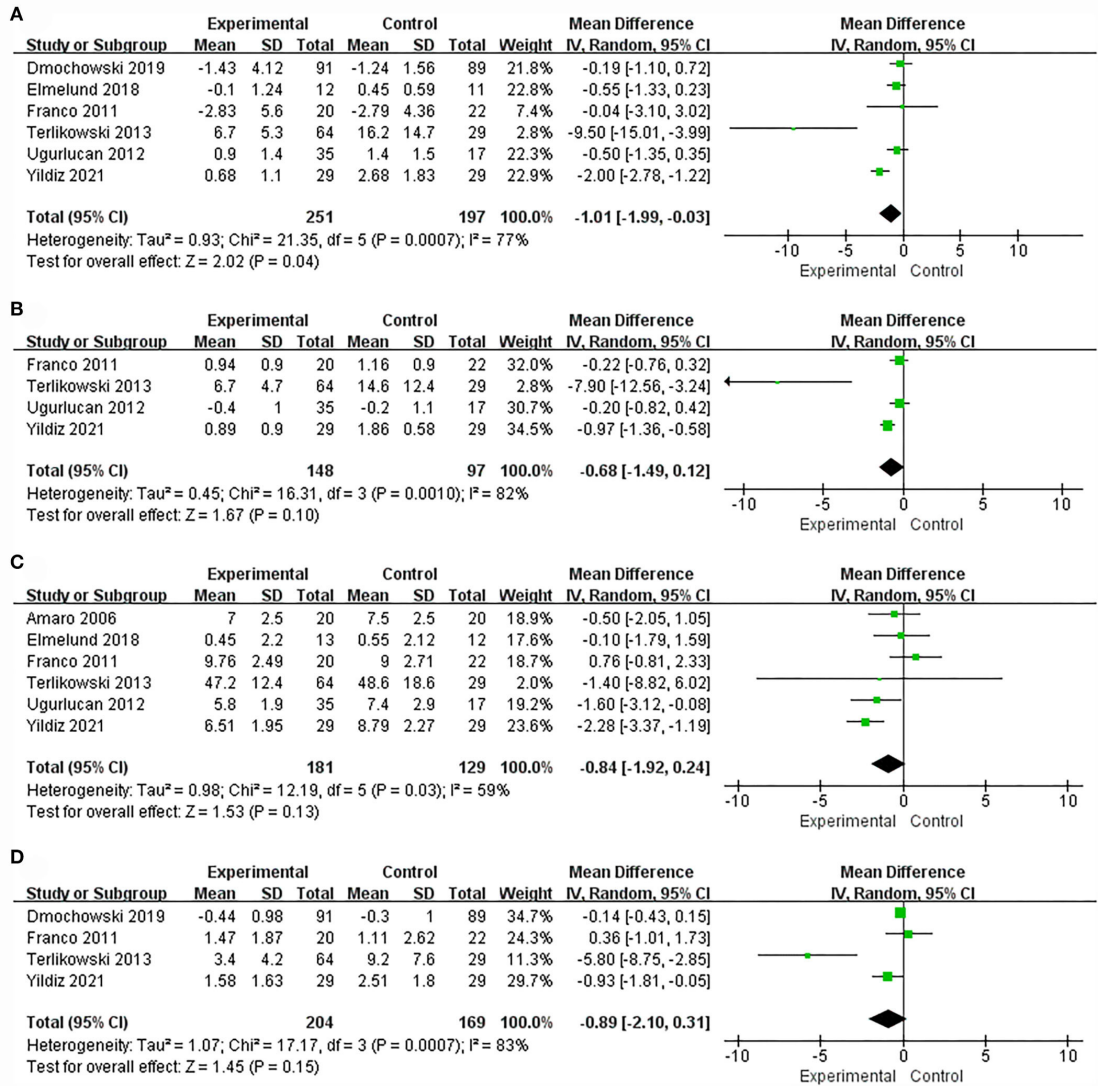
Forest plots showing the changes in bladder diary after IVES treatment. **(A)** Incontinence episodes; **(B)** Nocturia; **(C)** Daytime micturition; **(D)** Number of urine pads. SD, standard deviation; IV, inverse variance; CI, confidence interval; df, degrees of freedom.

Overall, six of the nine articles provided data on changes in the average number of episodes of urinary incontinence *per* 24 h, and the meta-analysis used a random effect model. The results showed that compared with the control group, the average number of episodes of urinary incontinence in the vaginal electrical stimulation group was significantly reduced (MD = −1.01, 95% CI = [−1.99, −0.03], *P* = 0.04; Figure 5A).

#### Nocturia episodes

Nocturia episodes refer to the number of times a person gets up and urinates at night after falling asleep. Four RCTs comprising 245 patients (148 and 97 in the experimental and control groups, respectively) described the change in the number of nocturia episodes per night. The results reported that there was no significant difference in the efficacy of IVES on nocturia episodes compared with the control group (MD = −0.68, 95% CI = [−1.49, 0.12], *P* = 0.10; Figure 5B).

#### Daytime micturition episodes

Daytime micturition episodes refer to the number times a person urinates during the daytime, and the normal range is usually from four to six times. Among the nine included studies, six articles provided the mean number of daytime micturition data. The results identified that there was no significant difference in reducing the number of nocturnal urination in the treatment group compared with the control group (MD = −0.84, 95% CI = [−1.92, 0.24], *P* = 0.13; Figure 5C).

### Number of urine pads

Changes in the number of urine pads used per day were also reported in randomized controlled trials. Four randomized controlled trials involving 373 patients (204 in the experimental group and 169 in the control group) described that compared with the control group, the results of the experimental group were not statistically significant (MD = −0.89, 95% CI = [−2.10, 0.31], *P* = 0.15; Figure 5D).

### King's health questionnaire

**Figure 6:**
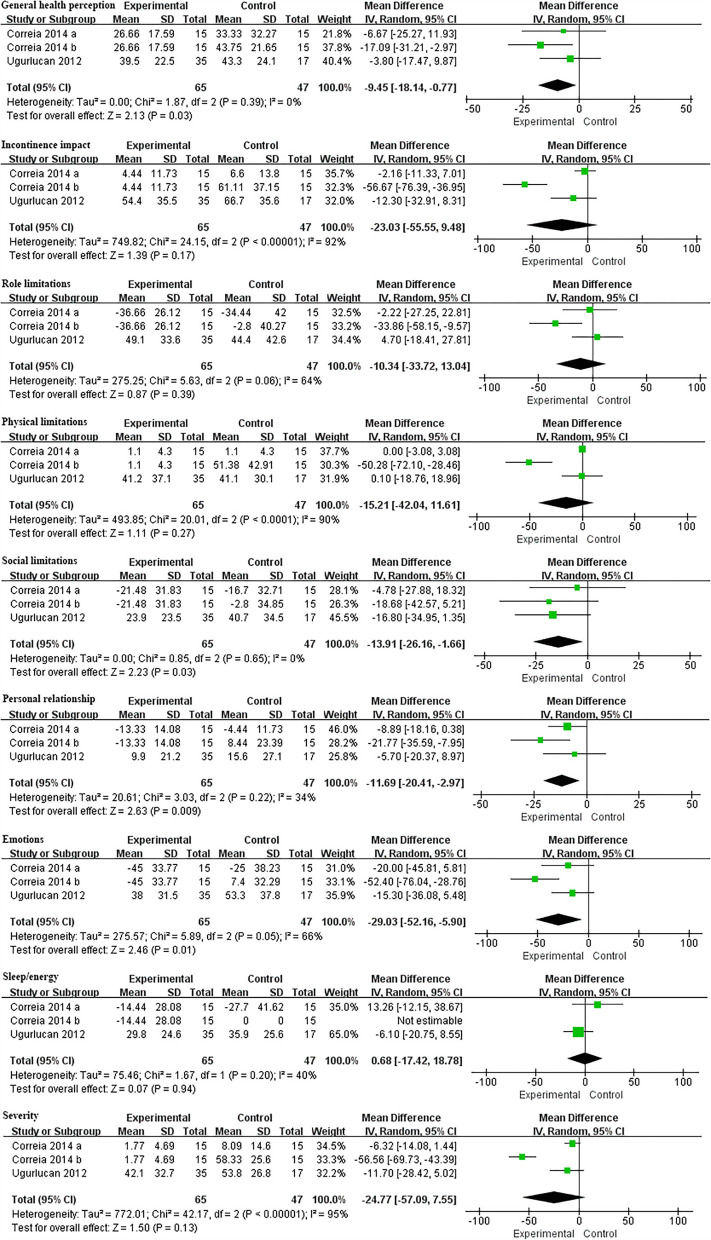
Forest plots showing the changes of various indexes in King's health questionnaire after IVES treatment. SD, standard deviation; IV, inverse variance; CI, confidence interval; df, degrees of freedom.

The King's health questionnaire is usually used as a quality-of-life evaluation index for female patients with urinary incontinence because it is easy to manage and increases the objectivity of patients' descriptions of subjective symptoms. Each item of this questionnaire is rated from 0 (best) to 100 (worst) (25). We found that a total of two RCTs mentioned the relevant indicators in the King's health questionnaire and the forest plot results are shown as follows. In the part of General health perception, the scores of the experimental group were significantly decreased compared with the control group, and the difference was statistically significant (MD = −9.45, 95% CI = [−18.14, −0.77], *P* = 0.03). There was no significant difference between the experimental group and the control group in the effects of incontinence impact, role limitations and physical limitations (Incontinence impact: MD = −23.03, 95% CI = [−55.55, 9.48], *P* = 0.17; role limitations: MD = −10.34, 95% CI = [−33.72, 13.04], *P* = 0.39; physical limitations: MD = −15.21, 95% CI = [−42.04, 11.61], *P* = 0.27). Besides, in the social limitations, personal relationship and emotions part, two RCTs identified that the improvement of the experimental group was significantly better than that of the control group (social limitations: MD = −13.91, 95% CI = [−26.16, −1.66], *P* = 0.03; personal relationship: MD = −11.69, 95% CI = [−20.41, −2.97], *P* = 0.009; emotions: MD = −29.03, 95% CI = [−52.16, −5.90], *P* = 0.01). In two studies comprising 97 participants (50 and 47 in the treatment and control group, respectively) reported sleep/energy and severity of urinary symptoms with an outcome respectively of MD = 0.68, 95% CI = [−17.42, 18.78], *P* = 0.94, and MD = −24.77, 95% CI = [−57.09, 7.55], *P* = 0.13. Unfortunately, the difference in these two results was not statistically significant. The forest plots of all relevant King's health questionnaire results are shown in Figure 6.

### Incontinence quality of life questionnaire

**Figure 7:**
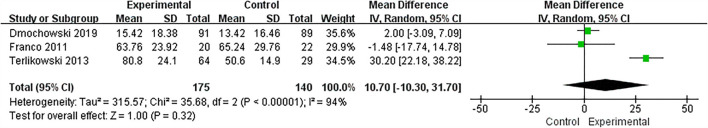
Forest plots showing the changes in I-QOL questionnaire scores after IVES treatment. SD, standard deviation; IV, inverse variance; CI, confidence interval; df, degrees of freedom.

Incontinence quality of life (I-QOL) questionnaire score is another quality-of-life assessment questionnaire for patients with urinary incontinence. Each question in the questionnaire reflects whether urinary incontinence is causing trouble, and the quantitative score ranges from one (completely) to five (never) (26). This questionnaire was mentioned in three studies and the result identified that the changes in the experimental group were not statistically significant compared with the control group (MD = 10.70, 95% CI = [−10.30, 31.70], *P* = 0.32, Figure 7).

### Pelvic floor muscle strength

**Figure 8:**
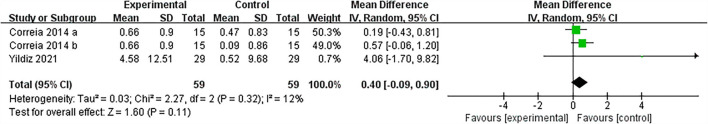
Forest plots showing the changes in PFM strength after IVES treatment. SD, standard deviation; IV, inverse variance; CI, confidence interval; df, degrees of freedom.

Pelvic floor muscle (PFM) strength measurement is another index to evaluate the outcome of urinary incontinence and a total of two studies mentioned this indicator. The results showed that compared with the control group, the IVES treatment could not significantly improve the pelvic floor muscle strength of participants, and the difference was not statistically significant (MD = 0.4, 95% CI = [−0.04, 0.84], *P* = 0.08, Figure 8).

### Subjective feeling

**Figure 9:**
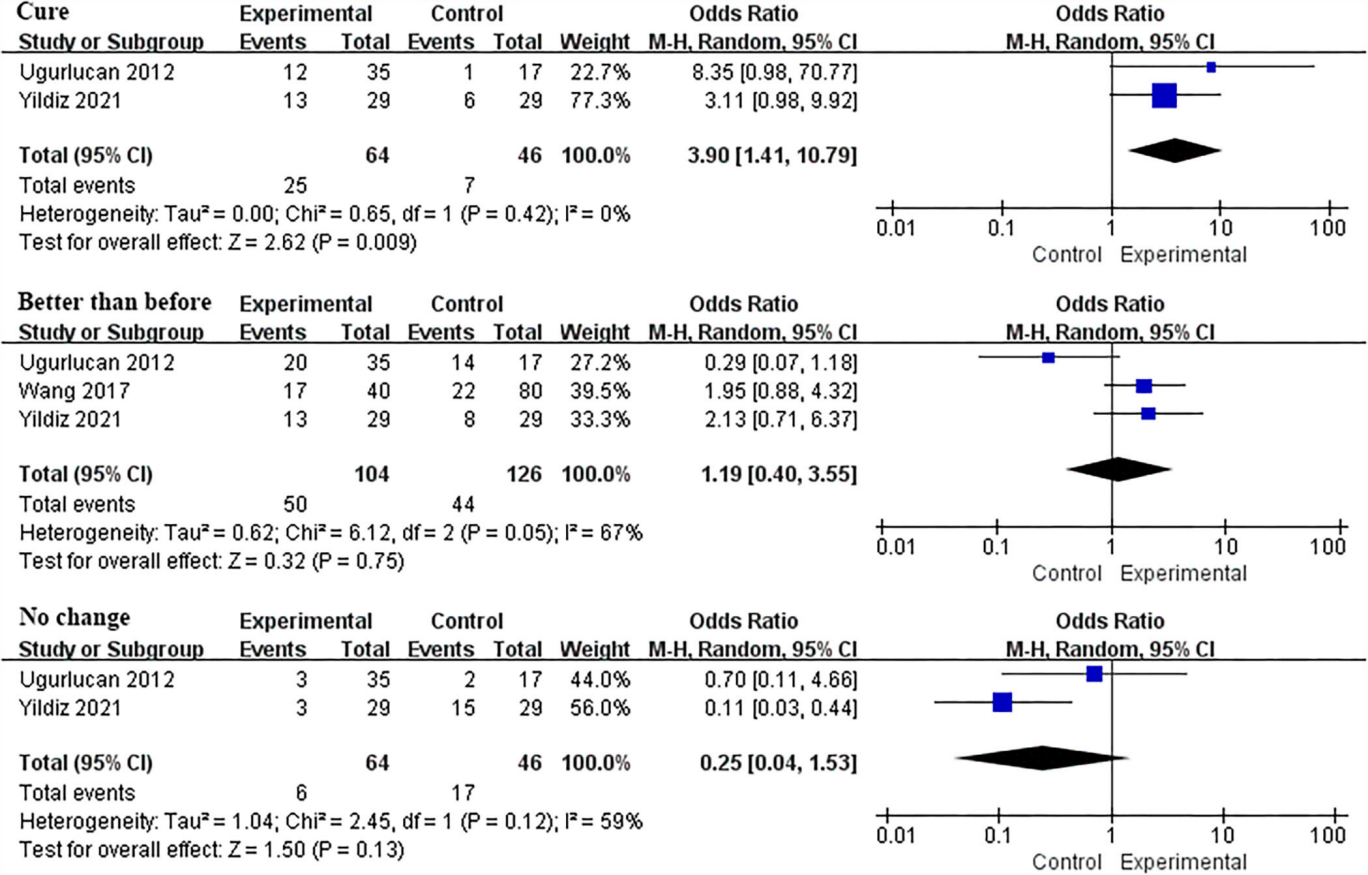
Forest plots showing the changes in patients' subjective feelings after IVES treatment. M–H, Mantel–Haenszel; CI, confidence interval; df, degrees of freedom.

The subjective feeling after treatment is usually used as a parameter to evaluate the effect of IVES. A total of three studies involving 230 participants (104 and 126 in the IVES and control groups, respectively) reported this data. The heterogeneity was high in the two parts of data in which the participants' responses were cure or no change. However, after deleting one of the studies (20), the heterogeneity was significantly reduced, so only the remaining two RCTs were analyzed. The results identified that the “cure” response rate of the IVES group was higher than the control group and the results were statistically significant (OR = 3.90, 95% CI = [1.41, 10.79], *P* = 0.009), while the “better than before” or “no change” response rate of the experimental group was not statistically significant compared with the control group (Better than before: OR = 1.19, 95% CI = [0.40, 3.55], *P* = 0.75; No change: OR = 0.25, 95% CI = [0.04, 1.53], *P* = 0.13; Figure 9).

### Subgroup analysis

**Figure 10:**
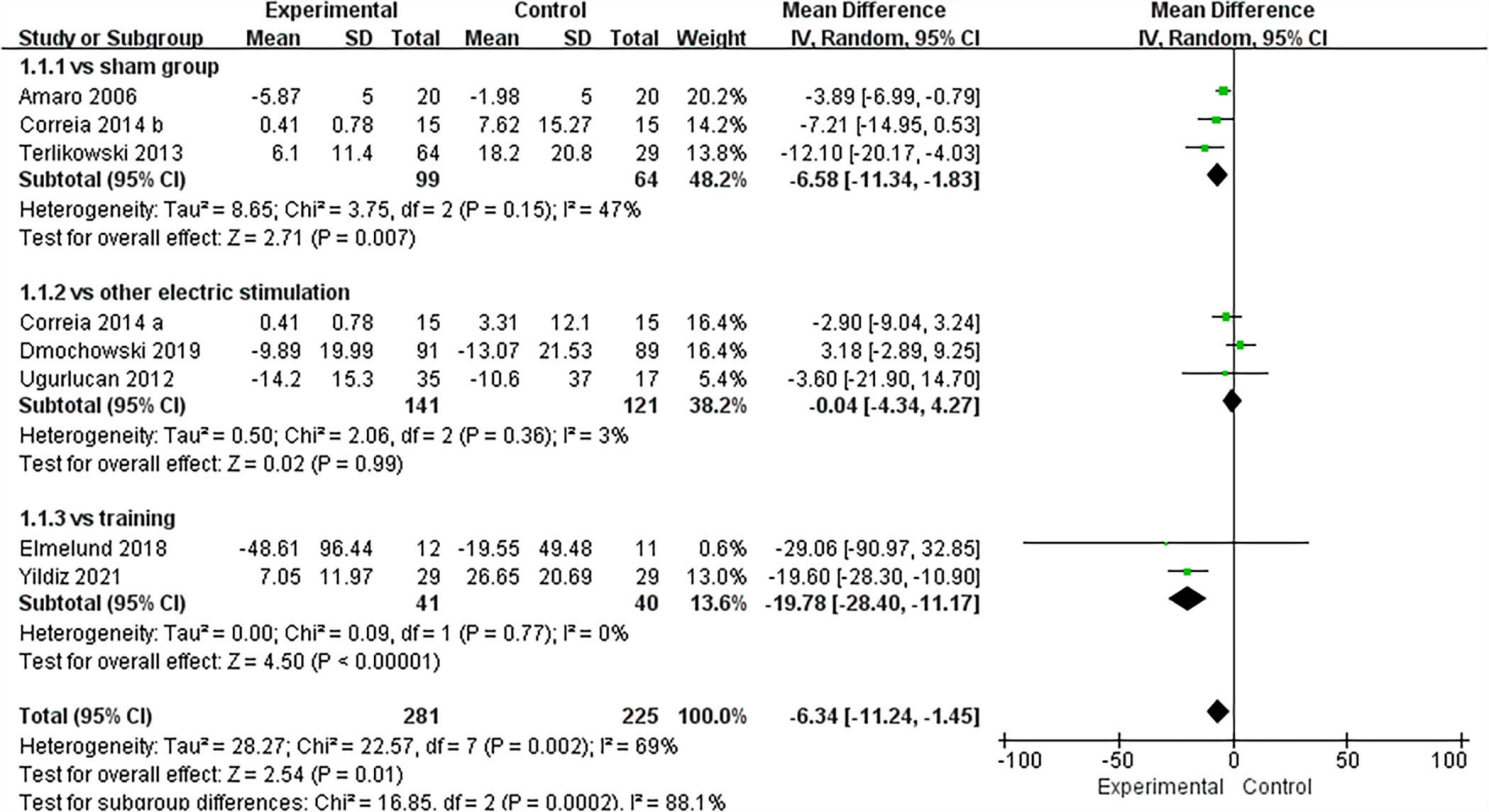
Forest plots showing the improvement of pad test in each subgroup. SD, standard deviation; IV, inverse variance; CI, confidence interval; df, degrees of freedom.

According to different experimental designs, we divided the included RCTs into three categories: IVES vs. sham group, IVES united training vs. training, and IVES vs. other electrical stimulation. At the same time, we performed a subgroup analysis of pad test, frequency of urinary incontinence, and the number of daily micturition. Through data analysis, it was found that IVES and the IVES united training can significantly reduce the weight of urine pad compared with the sham group and simple training group (IVES VS sham group: MD = −6.58, 95% CI = [−11.34, −1.83], *P* = 0.007; IVES united training vs. training: MD = −19.78, 95% CI = [−28.40, −11.17], *P* < 0.00001) while there was no significant difference between the IVES and other electrical stimulation groups (IVES vs. sham group: MD = −0.04, 95% CI = [−4.34, 4.27], *P* = 0.99; Figure 10). In the subgroup analysis of the urinary incontinence frequency, compared with the sham group, IVES can reduce the frequency of urinary incontinence, while IVES is not significantly improved compared with other electrical stimulation group or bladder training group. In addition, there was also no statistically significant difference among the three subgroups in the subgroup analysis of the number of daily micturition. The detailed results are shown in the Supplementary Figures S1, S2.

## Discussion

We conducted a systematic review and meta-analysis of nine RCT studies, including 657 participants, to evaluate the efficacy of IVES in the treatment of female urinary incontinence. The results identified that after treatment with IVES, the urine pad test and urinary incontinence frequency of patients were improved to a certain extent. In addition, the ratio of cure in the subjective feeling of patients in the experimental group was also better than that in the control group. Some indicators in the King's health questionnaire and I-QOL questionnaire had also improved, but there were still some other indicators' changes in the questionnaire that had no statistical significance. Besides, the improvement of PFM strength after the IVES treatment was also not satisfactory. The above results suggested that IVES may partially improve the symptoms of patients with urinary incontinence.

The clinical treatment methods for female urinary incontinence mainly include drug therapy, physical therapy, and surgical treatment (27–30). Among them, physical therapy has become the preferred treatment for many patients with urinary incontinence because of its relatively simple treatment, low medical cost, and almost no side effects and adverse reactions (31). Physical therapy mainly includes pelvic floor muscle training, bladder training, and nerve regulation (32–34). Electrical stimulation is a way of nerve regulation and it is to stimulate the afferent nerve, efferent nerve, or effector innervating the bladder by outputting appropriate current through the electrical stimulation device, so as to reduce the sensitivity of the bladder in patients and improve the state of overactive bladder (35, 36). At present, the common clinical electrical stimulation methods are to stimulate the common peroneal nerve, sacral nerve, and posterior tibial nerve *in vitro*, but there are few reports on the treatment of urinary incontinence by transvaginal electrical stimulation of the pudendal nerve.

Urgent urinary incontinence (UUI) and stress urinary incontinence (SUI) are two common types of UI. UUI refers to the behavior of bladder urinating without conscious control, which is usually secondary to cystitis, neurogenic bladder, and severe bladder outlet obstruction. SUI refers to the involuntary outflow of urine when the intra-abdominal pressure increases suddenly (cough, sneeze, laugh, exercise, etc.). SUI is common in women with multiple deliveries or postmenopausal women, which is caused by the weakening or loss of tension in the anterior vaginal wall and pelvic floor supporting tissue. Among the nine included studies, three studies mentioned the impact of IVES on UUI (Ugurlucan, Yildiz, Wang), and another three articles mentioned the impact of IVES on SUI (Correia, Terlikowski, Dmochowski). Ugurlucan's and Yildiz's research showed that IVES can significantly reduce the frequency of urinary incontinence and urine pad weight in UUI patients, and the subjective cure rate and satisfaction were also significantly improved. The research results of Correia, Terlikowski, and Dmochowski showed that in addition to improving the symptoms of urinary incontinence and subjective cure rate of patients, the improvement of IVES in patients with SUI was also reflected in PFM contraction intensity and pressure.

Our meta-analysis results found that IVES could improve a portion of symptoms of UI patients, which is consistent with the results of Correia et al. (16) and Franco et al. (17). Amaro et al. (18) and Elmelund et al. (24) found through their research that the control group and the experimental group achieved the same treatment effect, so they questioned the effectiveness of IVES, which may be caused by too few follow-up people (Amaro: 40; Elmelund: 27). Dmochowski et al. (21) and Wang (20) believed that the effect of the control group was better than that of the IVES group, which may be due to the fact that the control group was not treated with sham therapy but with other electrical stimulation methods. In addition, the research of Yildiz, Terlikowski, and Ugurlucan believed that although IVES had brought a certain degree of improvement and relief to patients' quality-of-life and symptoms, more research and longer follow-up time were still needed to evaluate the effect of IVES.

As a matter of interest, the subgroup analysis results showed that IVES and IVES united training could significantly reduce the weight of urine pads compared with the sham group and the simple training group. While compared with other electrical stimulation methods, the improvement of IVES was not obvious, and the difference was not statistically significant. These results suggested that we can consider using IVES combined training to treat female urinary incontinence. In addition, we also performed a subgroup analysis on the frequency of urinary incontinence and daily micturition, but we did not get statistically significant results.

In the process of data analysis, we found that some studies only provide sample median and interquartile range (IQR). Therefore, we, at last, used the formula to convert them into mean data and standard deviation to facilitate our statistics and comparison (37, 38).

This is the first meta-analysis to study the efficacy of IVES in the treatment of female urinary incontinence. Although the intention is novel and the included studies are high-quality randomized controlled trials, there are still some limitations. First, there are too many indicators to evaluate urinary incontinence, and the indicators used by different RCTs are not unified, so some indicators lack sufficient experimental data to support our analysis. Second, the application of IVES is not popular, so there are few relevant studies, which is not conducive to our subgroup analysis. Third, some indicators in the research are displayed through the median and interquartile spacing, and we must use the formula to convert the original data into mean and standard deviation to facilitate our final statistical analysis. This conversion process may bring some errors, which affect the final result. At last, our study did not report other indicators to evaluate urinary incontinence, such as urethral pressure, maximum bladder volume, and other questionnaires reflecting the quality of life of urinary incontinence, because only one randomized controlled trial reported these indicators, which is not conducive to our analysis of the data. In conclusion, we may need more unified indicators, more studies and results to help us evaluate the effect of IVES in the treatment of female urinary incontinence.

## Conclusion

This meta-analysis of nine randomized controlled trials showed that IVES could partially improve some indicators reflecting UI, such as lightening the quality of urinary pad in urinary pad test, reducing the frequency of urinary incontinence, increasing the proportion of cure in subjective evaluation, etc. However, there were still some indicators that did not improve significantly. In general, IVES is gradually becoming a popular choice for the treatment of female UI because of its effectiveness and convenience, but more experimental data are still needed to support our conclusion.

## Data availability statement

The original contributions presented in the study are included in the article/Supplementary material, further inquiries can be directed to the corresponding author/s.

## Author contributions

HY, XZ, FS, GT, JW, and ZZ study concept and design. HY, XZ, FS, JW, and ZZ analysis and interpretation of data and preparation of the manuscript. JW and ZZ critical revision of the manuscript. All authors contributed to the article and approved the submitted version.

## Funding

This work was supported by grants from the National Nature Science Foundation of China (Nos. 81870525 and 81572835) and Taishan Scholars Program of Shandong Province (No. tsqn201909199).

## Conflict of interest

The authors declare that the research was conducted in the absence of any commercial or financial relationships that could be construed as a potential conflict of interest.

## Publisher's note

All claims expressed in this article are solely those of the authors and do not necessarily represent those of their affiliated organizations, or those of the publisher, the editors and the reviewers. Any product that may be evaluated in this article, or claim that may be made by its manufacturer, is not guaranteed or endorsed by the publisher.

## Supplementary material

Supplementary Figure S1 Forest plots showing the improvement of urinary incontinence frequency in each subgroup. SD, standard deviation; IV, inverse variance; CI, confidence interval; df, degrees of freedom.

Supplementary Figure S2 Forest plots showing the improvement of the number of daily micturition in each subgroup. SD, standard deviation; IV, inverse variance; CI, confidence interval; df, degrees of freedom.
