# Supplementary figures and images for: The efficacy of intravaginal electrical stimulation (IVES) in treating female with urinary incontinence symptom from meta-analysis of nine randomized controlled trials
Source: Front Neurol. 2022 Sep 13;13:933679. doi: 10.3389/fneur.2022.933679 (PMC9514856; doi:10.3389/fneur.2022.933679)

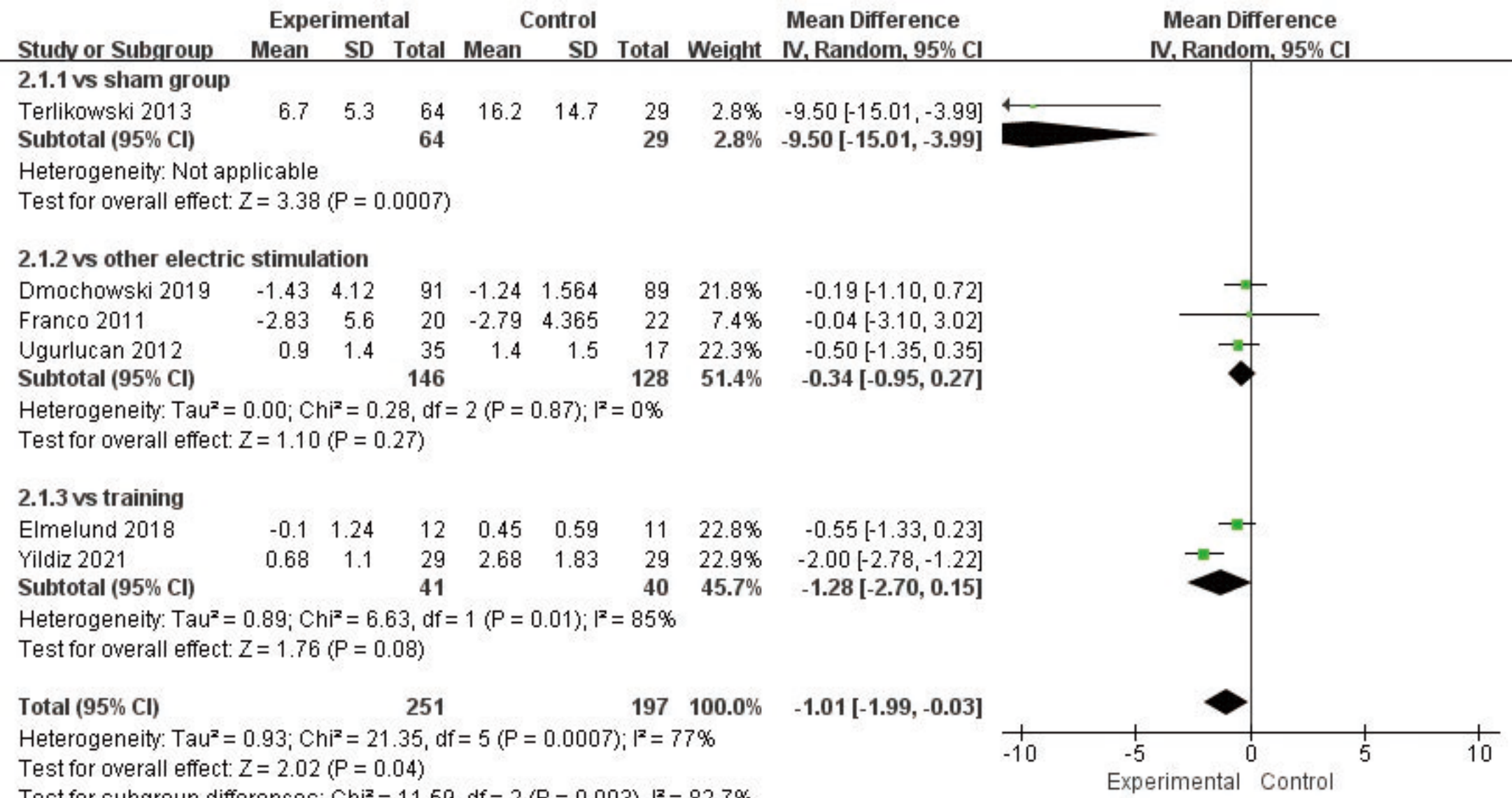

Supplement: Supplementary Figure S1 — Forest plots showing the improvement of urinary incontinence frequency in each subgroup. SD, standard deviation; IV, inverse variance; CI, confidence interval; df, degrees of freedom. [file Data_Sheet_1.PDF]

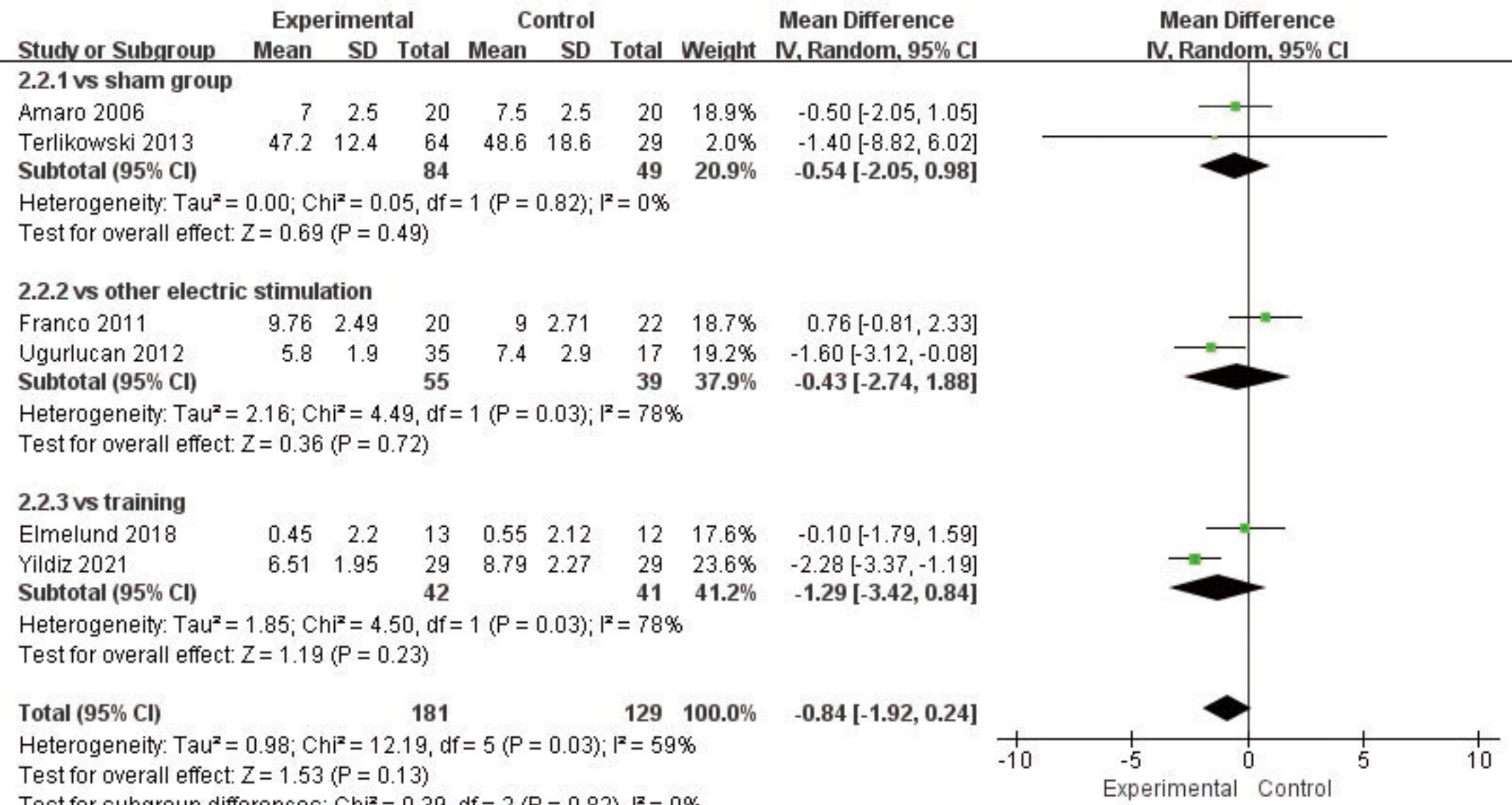

Supplement: Supplementary Figure S2 — Forest plots showing the improvement of the number of daily micturition in each subgroup. SD, standard deviation; IV, inverse variance; CI, confidence interval; df, degrees of freedom. [file Data_Sheet_2.PDF]
